# Potential of *TLR*-gene diversity in Czech indigenous cattle for resistance breeding as revealed by hybrid sequencing

**DOI:** 10.5194/aab-62-477-2019

**Published:** 2019-07-26

**Authors:** Karel Novák, Marek Bjelka, Kalifa Samake, Terezie Valčíková

**Affiliations:** 1 Department of Genetics and Breeding, Institute of Animal Science, Prague – Uhříněves, 104 00, Czech Republic; 2 Breeding company CHD Impuls, Bohdalec, 592 55, Czech Republic; 3 Department of Genetics and Microbiology, Charles University, Prague, 128 43, Czech Republic; 4 Department of Genetics and Breeding, Czech University of Life Sciences, Prague – Suchdol, Prague, 165 06, Czech Republic

## Abstract

A production herd of Czech Simmental cattle (Czech Red Pied, CRP), the conserved subpopulation of this breed, and the ancient local breed Czech Red cattle (CR) were screened for diversity in the antibacterial toll-like receptors (TLRs), which are members of the innate immune system. Polymerase chain reaction (PCR) amplicons of *TLR1*, *TLR2*, *TLR4*, *TLR5*, and *TLR6* from pooled DNA samples were sequenced with PacBio technology, with 3–5× coverage per gene per animal. To increase the reliability of variant detection, the gDNA pools were sequenced in parallel with the Illumina X-ten platform at low coverage (60× per gene). The diversity in conserved CRP and CR was similar to the diversity in conserved and modern CRP, representing 76.4 % and 70.9 % of its variants, respectively. Sixty-eight (54.4 %) polymorphisms in the five *TLR* genes were shared by the two breeds, whereas 38 (30.4 %) were specific to the production herd of CRP; 4 (3.2 %) were specific to the broad CRP population; 7 (5.6 %) were present in both conserved populations; 5 (4.0 %) were present solely for the conserved CRP; and 3 (2.4 %) were restricted to CR. Consequently, gene pool erosion related to intensive breeding did not occur in Czech Simmental cattle. Similarly, no considerable consequences were found from known bottlenecks in the history of Czech Red cattle. On the other hand, the distinctness of the conserved populations and their potential for resistance breeding were only moderate. This relationship might be transferable to other non-abundant historical cattle breeds that are conserved as genetic resources. The estimates of polymorphism impact using Variant Effect Predictor and SIFT software tools allowed for the identification of candidate single-nucleotide polymorphisms (SNPs) for association studies related to infection resistance and targeted breeding. Knowledge of *TLR*-gene diversity present in Czech Simmental populations may aid in the potential transfer of variant characteristics from other breeds.

## 1 Introduction

Resistance breeding is a prospective tool for the prevention of increases in infectious diseases in production populations of dairy cattle. This trend is considered to be a consequence of the increasing physiological load, unintended co-selection, and inbreeding associated with preferential breeding for milk production (Boichard et al., 2015). It also results from the emergence of new diseases (Purse et al., 2005) that are often resistant to standard antibiotic treatment.

In spite of the general adoption of genomic selection, an approach that targets causal genes might be more efficient in specific cases because the full available genetic variation is used. Disease-related breeding values are often underrepresented in combined breeding indices and the efficiency of current genomic selections might be disturbed by specific configurations in haplotype blocks (Abdel-Shafy et al., 2014).

The obvious targets for resequencing are components of the innate immune system (Boichard et al., 2015). In contrast to those of adaptive immunity they are fully determined in the germ line and are not formed ontogenetically (Kawai and Akira, 2010). A central role is played by the pattern-recognizing receptors (PRRs) that are activated by ligands originating in pathogenesis. The primary class of PRRs are the toll-like receptors, which are encoded by 10 gene paralogs denoted *TLR1*–*TLR10* in cattle (toll-like receptors; Jungi et al., 2011). The so-called antibacterial series, comprising proteins TLR1, -2, -4, -5, and -6, recognize molecules from the cell walls of gram-negative and gram-positive bacteria. Notably, TLR5 recognizes the protein flagellin from bacterial flagella.

Natural variation in the *TLR* genes of cattle has been characterized across the panel of world breeds (Jann et al., 2008; Seabury et al., 2010; Fisher et al., 2011). Many of the variants, particularly the non-synonymous ones that code for an amino acid (aa) change in the coded product, allow for prediction of the consequences to the function of the corresponding receptor. For example, the well-known mutation 9787C > T (c.2021) in the *TLR4* product is associated with a change in polarity in the crucial transmembrane part of the molecule, thereby affecting signal transfer (White et al., 2003). In accordance with expectations, this predicted functional change led to a shift in somatic cell count in cattle populations (Sharma et al., 2006; Beecher et al., 2010).

The importance of conserved historical populations of farm animals as a source of genetic diversity for resistance breeding is often acknowledged (FAO, 2007). Although the role of conserved populations in the protection of historical breeds is plain, gene pool erosion because of limited population sizes may significantly reduce their practical application in breeding; however, direct comparisons of the genetic richness in immunity genes with the modern production populations remain limited (Bilgen et al., 2016). For example, in historical Czech Red cattle previous studies reported high diversity in the major histocompatibility complex receptors (Hořín et al., 1997) and provided insight into the diversity of *TLR4* (Novák et al., 2017).

Screening for variations in antibacterial members of the *TLR* gene family was conducted for Czech Simmental (Czech Red Pied, CRP) cattle to explore their potential use in breeding with the goal of disease resistance. To estimate the value of historical breeds as genetic resources for CRP, two related conserved populations were studied.

The Czech Red Pied breed was developed in the territory of Bohemia and Moravia during the 19th century from the imported original Simmental cattle. Breed book registration of bulls of the Fleckvieh and Montbélliard breeds began in 2000; therefore, the current trend leads to the convergence of gene pools with the major Simmental breeds. For conservation purposes, a nucleus herd was formed in 2010 from 70 animals corresponding to the gene pool prevailing at the end of the 1990s (Mátlová, 2013).

The CRP breed is considered to be dual purpose. The female population consists of 212 000 cows, representing 63.3 % of the female Holstein population and 32.7 % of the total cow population in the country. The difference in milk production compared to the Holstein breed persists; the CRP breed produces 7137 kg yr${}^{-1}$ compared to 9426 kg yr${}^{-1}$ in Holsteins (Czech-Moravian Breeder's Corporation, summarized data of 2017). On the other hand, the milk fat and protein contents are reliably more favourable in CRP. Regarding the proportions of the population, 40–70 bulls are evaluated for breeding value every year.

The third studied population was the conserved population of Czech Red cattle. The breed is sometimes tracked back to the ancient Celtic cattle of Roman times, analogous to other red highland cattle breeds of central and western Europe such as Harz Mountain cattle and Salers; however, molecular data for independent conclusions have yet to be collected (Ludwig et al., 2016). Czech Red cattle prevailed until the 18th century in the territory of Bohemia and Moravia and presumably contributed to the formation of the Czech Simmental. The actual herd was restored in 1978 from only 14 cows and one bull with 50 % identity (http://www.fao.org/dad-is/en, last access: 15 July 2019). The current number of animals in the conserved population is approximately 250.

To avoid the necessity of validation of the revealed polymorphisms using individual genotyping reactions, the principle of hybrid resequencing was applied. Dataset noise was mostly suppressed by combining two technologies for population resequencing. In addition to polymerase chain reaction (PCR) amplicons that were prepared from pooled DNA samples and sequenced with PacBio technology with 3–5× coverage per gene per animal, the gDNA pools were sequenced with the Illumina X-ten platform at low coverage. In the next step, the functional consequences for the identified non-synonymous variants were predicted. Knowledge of gene variants occurring in the involved breeds allows for a careful transfer of information about their contribution to disease resistance from previous studies in other breeds.

## 2 Material and methods

### 2.1 Input DNA samples

The primary collection of DNA samples was from 150 bulls in the Czech Red Pied production population. DNA was prepared from cryo-preserved insemination doses using the MagSep tissue method (Eppendorf, Hamburg, Germany). Insemination doses of 100 µL were incubated with lysis buffer and 25 µL of proteinase K from the MagSep kit in the presence of 80 mM dithiothreitol at 55 ${}^{\circ }$C for 12 h with occasional vortexing prior to isolation.

The two conserved populations were characterized using DNA isolated from the aliquots of compulsory blood samples provided to the gene bank of the genetic resources programme. Thirty-five animals of the conserved CRP and 80 animals of CR (Czech Red) were included. One hundred microlitres of thawed blood was processed in silica membrane columns using the BloodPrep commercial procedure (Life Technologies, Carlsbad, CA, USA).

The concentration of the isolated DNA was determined fluorometrically on a microplate with SybrGold stain (Biotium, Fremont, CA, USA) and camera G:BOX Chemi XR5 (Syngene, Cambridge, UK) equipped with an EtBr filter under blue light excitation. Normalized genomic DNA (20 ng µL${}^{-1}$) was used as a template for PCR amplification of the exonic and flanking regions of all five antibacterial *TLR* genes. The amplification primers largely corresponded to those in previously published studies (White et al., 2003; Seabury et al., 2007; Seabury and Womack, 2008) and are listed in Table 1; the optimized PCR conditions are also listed. The PCR product yield was estimated according to the agarose electrophoresis control with GelRed (Biotium) fluorescent staining and a mixed sample was prepared containing all PCR fragments in equimolar concentration. The amplicon pool was purified on NucleoSpin columns (Macherey-Nagel, Düren, Germany) to remove unspent deoxynucleotide triphosphates, primers, and the short products of mis-primed PCR.

The pooled samples for low-coverage whole-genome sequencing were prepared from the population gDNA samples in equimolar concentrations. Purification was performed with the AMPure XP magnetic bead procedure (Beckman Coulter, Brea, CA, USA).

**Table 1:** Used PCR amplicons in antibacterial *TLR* genes and the optimized PCR conditions.

Gene	Fragment denotation	Amplicon start${}^{a}$	Ampliconend${}^{a}$	Productlength(basepairs)	Forwardprimerdenotation	Forward primer sequence 5${}^{\prime }$ → 3${}^{\prime }$	Reverseprimerdenotation	Reverse primer sequence 5${}^{\prime }$ → 3${}^{\prime }$	Annealingtemperaturein PCR(${}^{\circ }$C)
TLR1	T1_1	196	1361	1166	1_1F	ATGCCTGACATCCTCTCACT	1_1R	AGAACCTTGATCTGAGGAGGT	62/60
TLR1	T1_2	992	2186	1195	1_2F	TGACCCAGGAAATGAAGTCT	1_2R	CCGTGTTAATGTATTTCTGCTG	62/60
TLR2	T2_1	1	816	816	2_1F	TCCTGCTCCATATTCCTACG	2_1R	TGACTGTGTTTGACATCATGG	62/60
TLR2	T2_2	556	1223	668	2_2F	CTCATTCATTTATGGCTGGC	2_2R	GACCTGAACCAGGAGGATG	62/60
TLR2	T2_3	911	1726	816	2_3F	CGGAAGGAGCCTCTGACCAGGCT	2_3R	CATGGGTACAGTCATCAAACTC	62/60
TLR2	T2_4	1581	2354	774	2_4F	AGCATCCATCAGTGAAATGAG	2_4R	GGTAAGAAGGAGGCATCTGG	60/58
TLR2	T2_5	2206	2935	730	2_5F	AGTTTAACCCAGTGCCTTCC	2_5R	TGGAGTCAATGATGTTGTCG	62/60
TLR2	T2_6	2813	3248	436	2_6F	CCTACTGGGTGGAGAACCTC	2_6R	ACCACCAGACCAAGACTGAC	62/60
TLR4	T4_1	-3	657	661	4_1F	CCAGGGTATTTTGTTATGGCTGGAACAT	4_1R	TGTTTGCAAATGAACCTAACCA	62/60
TLR4	T4_2	4999	5382	384	4_2F	TCTTTGCTCGTCCCAGTAGC	4_2R	AAGTGAATGAAAAGGAGACCTCA	62/60
TLR4	T4_3	7941	9154	1214	4_3F	GGAGACCTAGATGACTGGGTTG	4_3R	AAGACAATGCGGATGTTGGT	62/60
TLR4	T4_4	8924	9596	673	4_4F	TTTCAAGGGGTGCTGTTCTC	4_4R	TGCACACATCATTTGCTCAG	64/62
TLR4	T4_5	9299	10 110	812	4_5F	AGCCCAGACAGCATTTCAC	4_5R	CTATAGGGCTCGCGTACCAC	62/60
TLR4	T4_6	9684	10 420	737	4_6F	GTCACTGTGCTCCTGGTGTC	4_6R	GCCGCAGGAGAGACTTCT	64/62
TLR5	T5_1	-3	638	642	5_1F	TTTGGGAAACGGAGGATAAG	5_1R	GCACCTTTGAGGCTGTGA	62/60
TLR5	T5_2	553	1241	689	5_2F	GCCTGCTTTTGATACTTTGG	5_2R	AGGTGTCCGCTATGTTCTCA	62/60
TLR5	T5_3	1065	1627	563	5_3F	TCCCTTACCTTCCAGCAGA	5_3R	AAGTTGGGGAAAACATTAGG	60/58
TLR5	T5_4	1495	2036	542	5_4F	GGCAGATTAGAGGGGAAAGA	5_4R	CCATCAAAGAAGCAGGAAGA	58/56
TLR5	T5_5	1927	2613	687	5_5F	TCACTCTCCCTTCTTCTCCA	5_5R	CAGACACTTGTTCCAGTCCA	60/58
TLR5	T5_6	2529	3231	703	5_6F	CCTCCAAGGGAAAACACTCT	5_6R	ATTGGCTGTAAGTGGGATGT	60/58
TLR5	T5_7	3153	3804	652	5_7F	TTTTCTTCCAAGCATTCCTA	5_7R	AGCCAGAGAGTTTGGGTACA	60/58
TLR5	T5_8	3623	4195	573	5_8F	GAAACCAGCTCCTCTCTCCT	5_8R	ATCTTTCTGCTGCTCCACAC	62/60
TLR5	T5_9	4059	4599	541	5_9F	AGACTTTGAATGGGTGCAGA	5_9R	TGGTAACTGGCGGAAATAAA	60/58
TLR5	T5_10	4536	5299	764	5_10F	GGAGCAGTTTCCACTTATCG	5_10R	ATTCTCATGCCGGTTTCTTT	58/56
TLR6	T6_1	ND	969	800	6_1F	ATTGAGAGTAATCAGCCAAT	6_1R	GTAAGGTTGGTCCTCCAGTG	60/58
TLR6	T6_2	707	1506	845	6_2F	ACTACCCATTGCTCACTTGC	6_2R	CTATACTCCCAACCCAAGAGC	62/60
TLR6	T6_3	1233	2077	845	6_3F	GACACACGCTTTATACACATGC	6_3R	CACTGACACACCATCCTGAG	62/60
TLR6	T6_4	1849	ND	800	6_4F	GCCAAGTATCCAGTGACGTG	6_4R	AATGGTGTTCTGTGGAATGG	62/60

${}^{a}$ In reference sequences FJ147090 (*TLR1*), EU746465 (*TLR2*), AC000135.1 (*TLR4*), EU006635 (*TLR5*), and AJ618974 (*TLR6*).

### 2.2 Resequencing

The pooled amplicons from the population DNA samples were sequenced with the PacBio technology (Pacific Biosciences, Menlo Park, California, USA). The libraries for sequencing were prepared in the GATC-Biotech sequencing core laboratory (Constance, Germany) using P4-C2 chemistry according to the manufacturer's procedure. One 120 min movie was obtained using the circular consensus sequencing (CCS) protocol on the PacBio RS II machine. The primary data in the h5 format were processed into the FASTQ format with Pacific Biosciences software (SMRT Analysis Software Suite).

The library for the technology of Illumina (San Diego, CA, USA) was prepared from the pooled gDNA sample in the core laboratory of Novogene (London, UK). Two rounds of pair-end 2×150 sequencing using the X-ten technology provided 60× coverage in total. After primary processing, the resulting files in the FASTQ format were used for variant identification.

### 2.3 Data processing

The FASTQ files containing reads obtained with the two technologies from all three populations were mapped to the reference sequences for all five genes. The reference sequences were FJ147090 for *TLR1*, EU746465 for *TLR2*, AC000135.1 for *TLR4*, EU006635 for *TLR5*, and AJ618974 for *TLR6 *(White et al., 2003; Seabury et al., 2007; Seabury and Womack, 2008). The sequences were downloaded from the nucleotide databases of the National Center for Biotechnology Information (NCBI) and European Bioinformatics Institute (EBI). The mapping algorithm Geneious Mapper implemented in the Geneious program package (Biomatters, Auckland, New Zealand) was used with the minimum mapping quality equal to 50. The alignments allowed us to detect structural variants using the Geneious algorithm at the maximum variant P=0.01 and the minimum occurrence in reads equal to 1.

The exported lists of variants in comma separated text file (csv) format were carried over to the coordinates of the UMD_3.1.1 assembly of the cattle genome. The single-nucleotide polymorphisms (SNPs) arising from the differences between the original reference sequences and the current genome assembly were added. The variants were filtered for clusters resulting from a read misalignment and were defined as more than 3 variants in a 25 nt stretch.

The filtered variants were compared between both platforms. Only the variants independently detected with both the Illumina and PacBio technologies were considered to be valid. Moreover, the consistency of the allelic frequencies based on the representation of the reads was used to distinguish the valid results. The presence of the variant in the EBI European Variation Archive (https://www.ebi.ac.uk/eva/?Home, last access: 15 July 2019) was treated as an additional criterion for SNP validation.

Haplotype structure was determined directly from the simultaneous occurrence of SNPs in the long reads provided by PacBio technology.

### 2.4 Population comparison

Nei's standard genetic distances (Nei, 1972) were calculated for the allelic frequencies generated by the next-generation sequencing (NGS) read representation and averaged for the two technologies. Graphic representation with un-rooted trees was generated with the neighbour joining algorithm and the FigTree program package (Rambaut and Drummond, 2010) for graph visualization.

### 2.5 Effect prediction

The list of the validated SNPs was submitted to the Variant Effect Predictor application (VEP) (McLaren et al., 2016) of the ENSEMBL database (https://www.ensembl.org/info/docs/tools/vep/index.html, last access: 15 July 2019). The SNPs were characterized for their potential effect on the *TLR* product according to the localization in the protein and the characteristics of the mutated codon. In the case of the non-synonymous mutations, functional changes were estimated by calculating the SIFT (sorting intolerant from tolerant) algorithm value (Sim et al., 2012) for the protein product. The SIFT score is the normalized probability that the amino acid change is tolerated. Substitutions with scores less than 0.05 are considered to be deleterious.

Both the reference and mutant protein sequences were sent to the SWISS-MODEL server of the University of Basel (https://swissmodel.expasy.org/, last access: 15 July 2019) for homology modelling of secondary and tertiary structure. The atom coordinates in the pdb format were visualized in the graphic system Yasara (Yasara Biosciences, Vienna, Austria). The graphic models were used to localize variable amino acids on the concave or convex side of the extracellular parts of the toll-like receptors and with respect to the functional domains involved in recognition, namely leucine-rich repeats (LRRs).

## 3 Results

### 3.1 Diversity

The variants found in the antibacterial *TLR* genes were mutually validated by their presence and frequencies in both datasets obtained with different sequencing technologies (Table 2; Novák, 2019). A total of 125 single-nucleotide change variants were detected in five genes, with 29 (23.2 %) of the variants being classified as previously unknown.

**Table 2:** Classes of *TLR*-gene variants found in the Czech Red Pied and Czech Red cattle populations.

Gene	Number	Novel	In coding		Population specificity${}^{b}$					
	of SNPs	SNPs	sequence${}^{a}$							
	found		Syn	NS	Both	CRP-	CRP	GR – both	CRP-	CR
					breeds	PR		breeds	GR	
*TLR1*	27	18	2	1	13	11	1	2	0	0
*TLR2*	34	1	12	13	8	20	0	3	3	0
*TLR4*	21	1	4	2	11	4	2	1	1	2
*TLR5*	20	3	2	1	17	0	1	0	1	1
*TLR6*	23	6	12	8	19	3	0	1	0	0
All genes	125	29	32	25	68	38	4	7	5	3

${}^{a}$ Syn – synonymous, NS – non-synonymous. ${}^{b}$ CRP – Czech Red Pied cattle, CRP-PR – production population of CRP, CRP-GR – conserved populations of CRP in genetic resources, CR – conserved Czech Red cattle.

The number of variants found in each population can be used to characterize their total diversity: 110 in the production population of CRP, 84 in the conserved CRP subpopulation, and 78 in the Czech Red cattle. Consequently, the diversity of the conserved populations of CRP and CR was close to the diversity of the far more abundant population of current CRP, representing 76.4 % and 70.9 % of its diversity, respectively.

**Figure 1:**
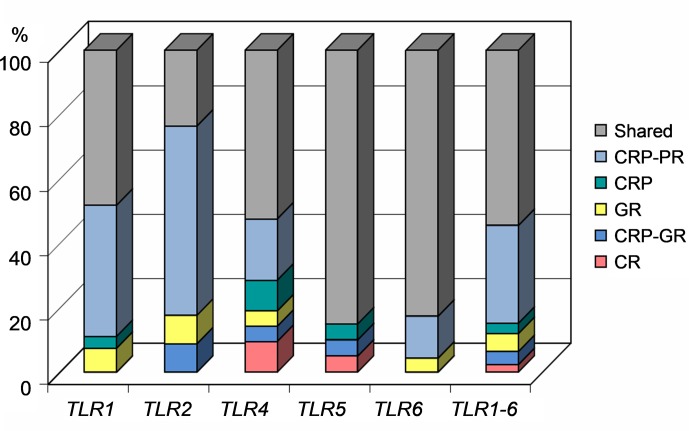
Proportion of gene variants according to the assignment to the studied cattle populations. CRP – Czech Red Pied cattle, CRP-PR – production population of CRP, GR – conserved populations in genetic resources, CRP-GR – conserved subpopulation of CRP, and CR – conserved Czech Red cattle.

All three populations – the production population of CRP, and the conserved populations of CRP and CR – shared 68 (54.4 %) polymorphisms (Table 2). Thirty-eight private SNPs (30.4 %) were confined to the production population of CRP; four (3.2 %) were characteristic of CRP in a broad sense, while only five (4.0 %) were associated strictly with the conserved subpopulation of this breed. Surprisingly, only three SNPs (2.4 %) were characteristic of the ancient Czech Red breed. The CR-specific variants comprised 1414C > T (intronic) and 8218A > C (missense) in *TLR4* and 25056C > T (downstream localization) in *TLR5*. Additionally, seven polymorphisms (5.6 %) were shared by both the conserved populations. The percentages of variants according to their population specificity, along with the individual genes, are shown in Fig. 1.

**Figure 2:**
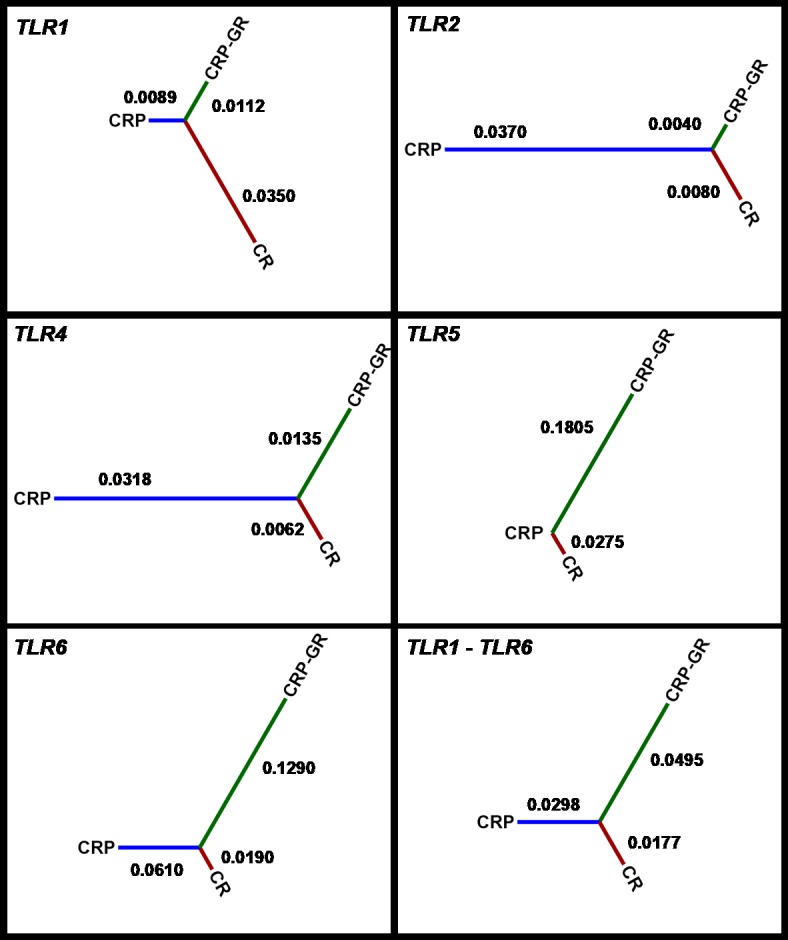
Inter-population distances based on all five *TLR* genes according to Nei's standard distance measure. CRP – production population of Czech Red Pied cattle, CRP-GR – conserved subpopulation of Czech Red Pied in genetic resources, and CR – conserved Czech Red cattle.

The allelic frequency derived from the read representation allowed for comparison of the population structure. The frequencies obtained with the two technologies were consistent in most of the SNPs detected and the mean value was used for subsequent interpretation. The inter-population distances based on all five *TLR* genes according to Nei's standard distance measure (Nei, 1972) between CRP, its conserved herd, and CR are visualized in Fig. 2.

### 3.2 Prediction of functional effects

The identified SNPs with breeding potential, i.e. the SNPs located in the coding sequence (CDS) or the promoter region, are presented in Table 3 along with predictions of their effects and their distribution among the populations.

In total, 57 of the found variants were located in the coding sequences, 32 of them being synonymous, and 25 non-synonymous. The ratio of non-synonymous mutations to the total number of CDS variants was 43.9 % (25 of 57) for all five genes, with the highest value, 52.0 %, in *TLR2*. In *TLR1*, intronic variants prevailed; therefore, only one variant with a moderate effect was reported by the VEP and SIFT programs and was associated with the conserved populations. Nevertheless, two intronic variants, not presented in Table 3, with a previously reported association with brucellosis (Prakash et al., 2014) were observed in all three populations. Their frequencies ranged from 0.054 to 0.143 and from 0.500 to 0.646 for SNPs 1575G > A (c.1380) and 1641A > C (c.1446), respectively.

**Table 3:** SNPs located to the coding sequence or to the promoter region of antibacterial *TLR*s present in the populations of Czech Red Pied and Czech Red cattle.

Chromosomal	gDNA	Nucleotide	SNP	CDS	Amino	Consequence	Impact	Variant frequency by		
position	position${}^{a}$	change	identifier	position	acid			population${}^{b}$		
					change			CRP-PR	CRP-GR	CR
*TLR1*										
6_59688857	798	C > T	rs43702940	603	F201F	synonymous	low	0.525	0.604	0.050
6_59687893	1762	G > A	rs210538093	1567	I523V	missense	moderate	0.629	0.767	0.050
6_59687558	2097	T > C	rs109456287	1902	F634F	synonymous	low	–	0.054	0.017
*TLR2*										
17_3953001	1044	T > C	rs68268249	186	N62N	synonymous	low	0.192	–	–
17_3952998	1047	G > T	rs55617172	189	E63E	synonymous	low	0.508	0.488	0.533
17_3952998	1047	G > A	novel	189	E63D	missense	moderate	–	–	0.143
17_3952985	1060	G > A	rs68268250	202	G68S	missense	moderate	0.171	–	–
17_3952732	1313	G > A	rs43706434	455	R152Q	missense	moderate	0.178	–	0.131
17_3952585	1460	G > A	rs110491977	602	S201N	missense	moderate	–	0.169	–
17_3952556	1489	G > A	rs43706433	631	V211I	missense	moderate	0.460	0.565	0.653
17_3952242	1803	G > T	rs68268253	945	R315R	synonymous	low	0.294	–	–
17_3952209	1836	T > A	rs68343167	978	H326Q	missense	moderate	0.286	–	–
17_3952177	1868	G > A	rs68343168	1010	R337K	missense	moderate	0.306	–	–
17_3951937	2108	A > G	rs68268256	1250	N417S	missense	moderate	0.244	–	–
17_3951879	2166	A > C	rs68268257	1308	G436G	synonymous	low	0.395	–	–
17_3951733	2312	C > T	rs210233457	1454	S485F	missense	moderate	–	0.048	–
17_3951555	2490	C > T	rs68268259	1632	F544F	synonymous	low	–	0.154	–
17_3951499	2546	G > A	rs68268260	1688	R563H	missense	moderate	0.291	–	–
17_3951480	2565	T > C	rs41830058	1707	H569H	synonymous	low	0.500	0.250	0.111
17_3951475	2570	G > C	rs41830058	1712	R571P	missense	moderate	–	–	0.029
17_3951408	2637	A > C	rs68268261	1779	A593A	synonymous	low	0.326	–	0.051
17_3951405	2640	G > T	rs68343169	1782	A594A	synonymous	low	0.289	–	0.050
17_3951336	2709	C > G	rs458702365	1851	Y617*	stop gain	high	–	–	0.021
17_3951192	2853	C > G	rs68268263	1995	H665Q	missense	moderate	0.100	–	–
17_3951162	2883	T > C	rs68343171	2025	H675H	synonymous	low	0.118	–	–
17_3951132	2913	T > C	rs68268264	2055	I685I	synonymous	low	0.140	–	–
17_3950973	3072	G > A	rs68268266	2214	E738E	synonymous	low	0.231	–	–
17_3950892	3153	C > T	rs68268267	2295	P765P	synonymous	low	0.177	–	–

**Table 3:** Continued.

Chromosomal	gDNA	Nucleotide	SNP	CDS	Amino	Consequence	Impact	Variant frequency by		
position	position${}^{a}$	change	identifier	position	acid			population${}^{b}$		
					change			CRP-PR	CRP-GR	CR
*TLR4*										
8_108828899	1	G > T	novel	–	–	5${}^{\prime }$–UTR	modifier	0.111	0.030	0.167
8_108829143	245	G > C	rs29017188	–	–	5${}^{\prime }$–UTR	modifier	0.212	0.467	0.471
8_108834032	5134	G > A	rs8193047	117	Q39Q	synonymous	low	–	0.022	–
8_108837116	8218	A > C	rs8193049	452	N151T	missense	moderate	–	–	0.207
8_108837831	8933	T > G	rs8193057	1167	G389G	synonymous	low	0.371	–	0.455
8_108838320	9422	C > T	rs8193060	1656	S552S	synonymous	low	0.231	0.397	0.416
8_108838685	9787	C > T	rs8193069	2021	T674I	missense	moderate	–	0.150	0.018
8_108838773	9875	C > A	rs447547035	2109	P703P	synonymous	low	0.018	–	–
*TLR5*										
16_27305951	23750	A > G	rs207872139	367	*123Q	stop lost	high	–	0.034	–
16_27304557	25144	T > C	rs55617187	1761	L587L	synonymous	low	0.592	0.425	0.521
16_27303858	25843	G > A	rs55617337	2460	D820D	synonymous	low	0.379	0.286	0.257
*TLR6*										
6_59706074	19281	G > A	rs43702941	640	D214N	missense	moderate	0.247	0.098	0.409
6_59706064	19271	G > C	novel	650	G217A	missense	moderate	0.343	–	–
6_59705939	19146	G > A	novel	775	V259M	missense	moderate	0.282	–	–
6_59705592	18799	T > C	rs68268274	1122	D374E	missense	moderate	0.376	0.355	0.321
6_59705362	18569	G > A	novel	1352	R451Q	missense	moderate	0.796	0.545	0.606
6_59705199	18406	T > C	rs798529324	1515	N505N	synonymous	low	0.683	0.446	0.441
6_59705137	18344	C > T	rs133754378	1577	A526V	missense	moderate	0.273	0.732	0.643
6_59705136	18343	G > A	rs136574510	1578	V526V	synonymous	low	0.273	0.054	0.143
6_59705094	18301	T > C	rs1116342462	1620	F540F	synonymous	low	0.714	0.639	0.500
6_59705084	18291	G > A	rs68268279	1630	V544I	missense	moderate	0.797	0.578	0.325
6_59705070	18277	C > A	rs1117717951	1644	S548S	synonymous	low	0.599	0.500	0.489
6_59704995	18202	G > A	rs482969002	1719	K573K	synonymous	low	0.247	0.421	0.230
6_59704949	18156	A > G	rs207882984	1765	I589V	missense	moderate	0.700	0.767	0.417
6_59704899	18106	C > T	rs378853146	1815	L605L	synonymous	low	–	0.119	0.077
6_59704764	17971	A > G	novel	1950	E650E	synonymous	low	0.622	–	0.729
6_59704692	17899	C > T	rs209572763	2022	H674H	synonymous	low	0.814	–	0.861
6_59704674	17881	C > T	novel	2040	A680A	synonymous	low	0.742	–	0.735
6_59687558	765	T > C	rs109456287	2100	F700F	synonymous	low	0.130	0.055	0.079
6_59687555	762	T > C	rs207586910	2102	V701V	synonymous	low	0.416	–	0.442
6_59687528	735	G > A	novel	2130	E710E	synonymous	low	0.444	–	0.132

${}^{a}$ Positions of SNPs are determined in the reference sequences FJ147090, EU746465, and AC000135.1 for *TLR1*, *TLR2*, and *TLR4*, respectively. In *TLR5* and *TLR6*, the positions are relative to gene starts in UMD_3.1.1. ${}^{b}$ CRP-PR – production population of Czech Red Pied cattle, CRP-GR – conserved populations of CRP in genetic resources, and CR – conserved Czech Red cattle.

Most of the non-synonymous nucleotide changes, which are potentially important for breeding, were concentrated in *TLR2* and *TLR6*. Of 13 missense *TLR2* variants, 12 were predicted to exert a moderate effect, and only one change with a predicted high effect (Y617*) was found in the conserved Czech Red cattle.

In variants of *TLR4 *(Table 3), four changes with low effect and four changes with moderate effect were found, of which T674I was associated with conserved populations and N151T with CR. Four intronic variants with known functional impacts (Ruiz-Larrañaga et al., 2011) are not listed among the CDS variants in Table 3. They were 5087A > G (rs8193046), 7999A > G, c.94–24A > G (rs8193046), and c.261–28G > A (rs43578100) with frequencies ranging from 0.246 to 0.553 in all three populations. Another active intronic variant, c.94–867A > G (rs43578097), was found only in the CRP production herd with a frequency of 0.182.

In spite of considerable total variation in the *TLR5* gene, only two mutations were associated with a small predicted effect and one with a large predicted impact; however, the stop-loss change *123Q (rs207872139), with a high expected impact, is confined to the conserved CRP population.

In contrast to *TLR5*, variability in the *TLR6* gene yielded 12 synonymous SNPs with a predicted low effect and eight amino acid substitutions associated with a moderate effect. Only one of the synonymous mutations, rs378853146, was restricted to the conserved populations.

The SIFT value, which quantifies the expected impact, dropped to 0.2 or below in four amino acid changes in *TLR2*, namely E63D (SIFT value 0.10), R571P (<0.01), R563H (<0.01), and Y617* (<0.01), and in the R451Q (0.20) substitution in TLR6; however, even the additional 21 variants in the studied gene set characterized as causing changes with moderate impact should be considered innate immunity factors. In addition, the change *123Q in TLR5 was classified as having a high impact by the VEP program alone.

Summing up, only 5 of 45 variants that were predicted to have effects on the toll-like receptors function were restricted to the conserved populations.

## 4 Discussion

### 4.1 *TLR*-gene variation in the studied herds

The study demonstrated considerable variation in the five members of the *TLR* family with antibacterial function in the current Czech Simmental population (Czech Red Pied cattle), in its conserved subpopulation representing the original gene pool, and in the presumably related historical breed Czech Red cattle.

Consistency with available data on the general diversity in cattle in the EBI variant database demonstrates the reliability of the hybrid sequencing (Koren et al., 2012). In this scheme, two different NGS technologies are combined to identify and eliminate the systemic faults of each. The technique of hybrid sequencing was originally suggested for obtaining correct de novo assemblies (Koren et al., 2012). This approach allows for avoidance of disambiguities originating from duplicates and large rearrangements. The principle is applicable to the validation of discovered SNPs, helping to avoid single-purpose genotyping reactions, a costly and laborious step in the discovery of polymorphisms. The application of this approach on a small scale for the description of variability in individual genes is well justified.

Nevertheless, the comparatively high proportion of novel SNPs observed (23.2 %) compared with the limited number of novel SNPs reported in an analogous study (Bilgen et al., 2016) for the Holstein population (only 4 novel SNPs of 274 for three *TLR* genes and 3 of 45 for *TLR2*) was surprising but can be ascribed to the attention focused on the Holstein population.

It should be noted that many of the observed polymorphisms have been previously reported, mostly for meat breeds, in the panel of 26 world breeds, namely 798C > T and 1762G > A in *TLR1*, 513C > T, 1047G > T, 1313G > A, and 2565T > C in *TLR2*, and 855G > A in *TLR6* (Seabury et al., 2007; Seabury and Womack, 2008; Seabury et al., 2010). The polymorphisms 9787C > T in *TLR4* and 855G > A in *TLR6* were represented in a panel of 16 European breeds at frequencies of 0.12 and 0.52, respectively (Mariotti et al., 2009).

Application of the PacBio technology provides in addition long reads (up to 1200 bp in the present work) that allow direct phasing of located SNPs. The data obtained in the current work are thus expected to contribute to the precision of the previously studied haplotype structure of the *TLR* genes (White et al., 2003; Seabury et al., 2010; Ruiz-Larrañaga et al., 2011; Bilgen et al., 2016; Novák et al., 2017). This approach preliminarily revealed as many as 15 haplotypes in amplicon T2_1 of *TLR2* in the studied populations, which is consistent with earlier reports of high haplotype numbers at this locus (Seabury et al., 2010; Bilgen et al., 2016).

### 4.2 Distinctness of conserved populations

Inclusion of the conserved subpopulation of the Czech Red Pied cattle, reflecting the genetic structure before 2000, facilitated the detection of selection trends in CRP. The diversity found in the conserved subpopulation of CRP, representing 75.4 % of the diversity of the main production population, demonstrates that gene erosion due to intensive breeding for the production traits has not occurred over the last 2 decades.

Significantly, only five SNPs were specific for the conserved historical population compared with 38 SNPs that were specific for the modern population. The low diversity of the conserved CRP population may have reflected the small number of sampled animals (n=30). In addition, the total size of the conserved CRP, which is approximately 70 animals, is close to the minimum effective population. The plausible conclusion is that after opening the breeding population to imported animals of the Fleckvieh and Montbélliard breeds, the consequent enrichment of the local Simmental population led to an increase in the available diversity in innate immunity genes, regardless of preferential breeding for utilitarian traits.

Notably, the allelic richness of the historical population of the Czech Red cattle after two historical bottlenecks remained comparable with the richness of the large population of CRP; however, the distinctness of the CR breed was characterized by only three breed-specific alleles when compared to both Simmental populations. This indicates that breed-specific diversity due to the ascribed ancient origin of the breed is not visible at the level of *TLR*-gene variants; moreover, the source of the breed-specific alleles might be admixture from the group of breeds that were used in the process of breed revitalization after a population bottleneck in 1987. This group of breeds included, in addition to Czech Simmental, Polish Red, German Mountain Red, Angler, and Ayrshire cattle. Full reconstruction of the specific admixture is possible on the basis of haplotype assignment (Wang et al., 2017).

The average allelic frequency-based differences among the studied populations were moderate, as characterized by Nei's distances. They corresponded to the previously demonstrated relatedness of Czech Red Pied and Czech Red cattle, as estimated using microsatellite polymorphisms in the context of other central European breeds (Čítek et al., 2006; Zaton-Dobrowolska et al., 2007).

The pattern of inter-population distances for different *TLR*s was consistent only between *TLR2* and *TLR4*. On the other hand, similarity was not observed between the polymorphisms of *TLR1* and *TLR6*, in spite of their identical localization on chromosome 6. The variation in distances in different *TLR*s must be partially assigned to the low number of SNPs available at individual loci.

The calculated inter-population distances did not support the distinctness of the historical breed CR, consistently with private allele occurrence. It should be noted that inter-varietal differences in bovine *TLR* polymorphisms are not generally high. For example, specialized beef and dairy breeds could not be differentiated despite an average polymorphism density of 1 marker per 158 bp in the *TLR* genes (Fisher et al., 2011).

The role of historical populations as a source of new functionally important alleles for breeding seems to be moderate; however, some of the variants restricted to the historical populations might be recruited to resistance breeding. The polymorphism H326Q in *TLR2*, which was identified as promising by Jann et al. (2008), was absent from the Holstein population but was observed in autochthonous Turkish breeds (Bilgen et al., 2016). In the case of CRP, this polymorphism is already present in the production herd. The 8218A > C polymorphism (N151T) restricted to CR might be a candidate for introgression after evaluation of its impact. Notably, the non-synonymous polymorphism H665Q, present in the Czech Red Pied population at 10 % frequency, is considered specific for the *Bos indicus* population (Jann et al., 2008).

### 4.3 Indications for functional consequences applicable in breeding

#### 4.3.1 Functionally relevant polymorphism

As shown in protein modelling that included 280 variable sites in all bovine *TLR* genes, up to 32 % of the aa substitutions affected protein function (Fisher et al., 2011). Consequently, a comparable proportion of functional changes could be assumed for the studied populations. The observed value of 20 % (5 of 25) according to VEP and SIFT predictions of high effects is within the expected range.

#### 4.3.2 *TLR1* polymorphisms

In spite of a limited number of mutations found in *TLR1* in the present work, they may contribute to resistance breeding. The non-synonymous mutation 1762G > A, present in all three populations, leads to a substitution I523V that disturbs the transmembrane region of the receptor (Russell et al., 2012). Accordingly, a higher susceptibility to bovine tuberculosis has been demonstrated for the AA and AG genotypes in Holstein cattle (Sun et al., 2012). On the other hand, the synonymous 798C > T (F201F) was tested with a negative result (Sun et al., 2012).

Synonymous 2097T > C (F634F), which was found to be limited to the conserved populations in our work, is presumably linked with 2463C > T in the 3${}^{\prime }$–UTR (untranslated region), exerting an effect on acute mastitis (Russell et al., 2012). Unfortunately, both active SNPs reported by Russell et al. (2012), namely -79T > G and 2463C > T, were out of the amplicon range and their presence was not directly observed.

In view of the epidemiological and economic importance of brucellosis, it is worth mentioning that two intronic mutations, 1575G > A (rs454341370) and 1641A > C (rs135207279), affecting this disease (Prakash et al., 2014) were present at frequencies permitting both testing in association studies and immediate use in selection. The absence of an effect on brucellosis in the otherwise active missense mutation 1762G > A (Prakash et al., 2014; Russell et al., 2012; Sun et al., 2012) suggests that the causal mutations are out of the screened range, presumably in the regulatory region of *TLR1,* and that linkage in the haplotype structure is critical for observation of the effect.

#### 4.3.3 *TLR2* polymorphism

Based on high diversity in *TLR2* and matches with the polymorphisms in other populations, the possibility of breeding applications is higher for this gene than for other *TLR* genes surveyed. Jann et al. (2008) predicted the functional consequences of the *TLR2* variants by defining the protein regions that show the signatures of positive selection, based on interspecific sequence comparison. The identified region corresponds to the domains participating in recognition spanning from aa 260 to 360. Of the known substitutions L227P, H305P, and H326G located in this region, H326G (rs68343167) is considered to be potentially the most efficient one because of the type of aa change; moreover, in human TLR2 this domain is crucial for ligand binding. Similarly, Bilgen et al. (2016) characterized the functional consequences of aa substitutions in TLR2 using exact localization in protein models.

**Figure 3:**
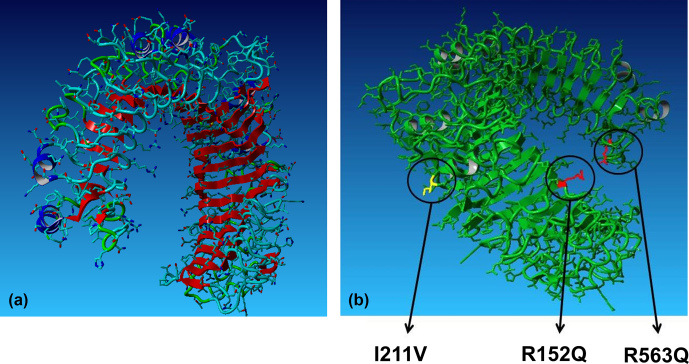
**(a)** Extracellular part of TLR2 with leucine-rich repeats indicated in red. **(b)** Placement of functionally relevant amino-acid substitutions in TLR2 found in the Czech indigenous cattle breeds.

Applying this information to the present purpose, most of the aa changes found in the studied populations could be placed in protein domains. E63D (in CR only) is located in LRR1, R152Q (shared by the three populations) in LRR5, I211V (shared polymorphism) in LRR7, H326G, and R563H (restricted to the CRP production herd) in LRR20, and H665Q (in the CRP production herd) is located in the highly conserved toll/interleukin-1 receptor (TIR) homology region. Positioning of these substitutions in functionally important regions increases the probability of detecting health effects in anticipated population studies. Notably, the functional effects of 2546G > A (R563H) disturbing LRR20 have been repeatedly predicted (Bilgen et al., 2016; Seabury et al., 2010; Fisher et al., 2011). The polymorphism H326G was observed in the studied populations and represents a potential target for breeding. Placement of the non-synonymous R152Q, I211V and R563H in LRRs of the extracellular part of the TLR2 protein is illustrated in Fig. 3.

Accordingly, the predicted effects found support on the population level. The E63D substitution in exon 2 of the *TLR2* gene was reported to be associated with susceptibility or resistance to tuberculosis in cattle and was suggested for exploitation in breeding upon validation (Bhaladhare et al., 2016). On the other hand, synonymous E63E in the same protein position was uniformly distributed across all studied populations with frequencies of approximately 50 % (Table 3) facilitating potential association studies.

The change of 513C > T in the 5${}^{\prime }$–UTR increases susceptibility to paratuberculosis (PTB) (Fisher et al., 2011), while 1047G > T (E63D) and 1313G > A (R152Q) disturbing LRR1 and LRR5, respectively, increase susceptibility to bovine tuberculosis (Bilgen et al., 2016; Bhaladhare et al., 2016). The latter SNPs are components of the *TLR2* haplotype that enhances resistance to PTB (Ruiz-Larrañaga et al., 2011).

Surprisingly, the C allele of the synonymous change 2565T > C (His569His) was associated with susceptibility to PTB in both the homozygous and heterozygous state (Koets et al., 2010). Accordingly, allele C is a part of the haplotype susceptible to PTB together with the neighbouring SNP rs6826825 (Phe550Phe) (Ruiz-Larrañaga et al., 2011; Koets et al., 2010). On the other hand, allele T is a part of a favourable combination of six genes for resistance to PTB (Juste et al., 2018).

Testing the complete haplotypes instead of individual polymorphisms is an approach that improves the resolution power of association studies (Ruiz-Larrañaga et al., 2011; Abdel-Shafy et al., 2014). Consequently, a risk haplotype of *TLR2* for *Mycobacterium avium* ssp. *paratuberculosis* (MAP) infection CAGGCCC composed of c.455G > A, c.457T > C, c.602G > A, c.631A > G, c.1049C > T, c.1632C > T, and c.1707T > C has been identified (Ruiz-Larrañaga et al., 2011). The general character of this result is illustrated by the fact that six of these polymorphisms were also present in the studied populations (Table 3).

Besides, the ability of *TLR* products to form functional heterodimers (Huang et al., 2011) and to interact with other innate immunity factors should be considered in estimates of gene variants. In a recent study, Juste et al. (2018) reported combinations of polymorphisms in a set of six innate immunity genes that contributed to PTB resistance in the Holstein breed. Polymorphism R571P (rs41830058) in TLR2, a part of the most favourable polymorphism combination, was also observed in the ancient CR population.

Some of the SNPs in *TLR2* were previously validated for their effects on disease resistance but were absent in the studied populations; therefore, their potential cannot be used in the current breeding scheme. They include the SNP resulting in I680V in the conserved TIR region that is associated with the MAP resistance (Mucha et al., 2009), c.1903T > C in which the C allele is associated with MAP resistance (Koets et al., 2010), and T385G, which is reported to affect mastitis incidence in Holstein, Simmental, and Sanhe cattle (Zhang et al., 2009).

#### 4.3.4 *TLR4* polymorphism

A functionally important SNP detected in *TLR4* of the studied populations, c.-226G > C (rs29017188), is localized in the 5${}^{\prime }$–UTR region and is supposed to alter *TLR4 *expression (Sharma et al., 2008). This change may affect the binding of corresponding transcription factors c-Ets-1, MZF1, and ADR1 (Sharma et al., 2008). The susceptibility to MAP infection conferred by the C allele (Ruiz-Larrañaga et al., 2011) raises the possibility that this mutation can be exploited in breeding.

The change 9787C > T (c.2021, T674I), which is located in the transmembrane region of the receptor, is well known for its effect on susceptibility to PTB (Fisher et al., 2011) and its positive effect on milk protein and fat contents (Beecher et al., 2010); however, the CRP population was found to be already monomorphic for the favourable C allele, although variation still persists.

The high frequencies that were discovered in five functionally relevant intronic variants of *TLR4* (Ruiz-Larrañaga et al., 2011) may be advantageous for phenotypic evaluation of these variants and potential use in selection. The effect of the intronic variant 5087A > G (rs8193046) with respect to PTB has been independently observed by Kumar et al. (2019) in a multi-breed population. In parallel, this effect has been demonstrated for the synonymous mutation 9422C > T (rs8193060), also found in the present study at convenient frequencies ranging from 0.231 to 0.416.

The effects of the mutation 5087A > G seem to be multilateral since an association with keratoconjunctivitis and calving ease has been reported (Mullen et al., 2018). This observation is not surprising in view of the range of known non-immune functions of toll-like receptors (Anthoney et al., 2018). In the particular case of calving ease, the effect of TLR4 variation might reflect a role in the inflammatory response and myometrial signalling before parturition, which is mediated by the MyD88/TRAF6/NF-κB pathway (Lim et al., 2017). The intronic localization of the SNP 5087A > G together with its multi-faceted effects points again to the importance of linkage in the haplotype structure for the detection of phenotypic associations.

Unfortunately, the *TLR4* alleles reported to be associated with the resistance to brucellosis (c.10C > T and c.399C > T) were not identified in the studied populations.

#### 4.3.5 *TLR5* polymorphism

The aa polymorphisms R262H and F643L in TLR5, previously associated with a predicted functional impact (Fisher et al., 2011), were not found in our populations; however, the R125* mutation, reported as a candidate polymorphism for MAP resistance (Fisher et al., 2011), may be identical to *123Q (rs207872139) observed in our study. This was interpreted by the SIFT algorithm as a stop-loss mutation with significant functional consequences.

#### 4.3.6 *TLR6* polymorphism

Notably, mutation 855G > A (D214N) in *TLR6*, reported to increase the susceptibility to PTB (Fisher et al., 2011), was present in all populations at frequencies ranging from 0.098 to 0.409. On the other hand, none of a further three polymorphisms with ascribed functional changes, namely L43R, R87G, and F494I (Seabury et al., 2010; Fisher et al., 2011), were identified. Nevertheless, an additional seven aa substitutions with predicted moderate effects were found in TLR6 in the studied populations and are worth including in association studies.

## 5 Conclusions

Hybrid sequencing of population samples combining long-range amplicon sequencing with PacBio technology and direct gDNA sequencing with Illumina HiSeq technology is a fast and reliable approach to a survey of total variation in target genes. Using this approach, the scope of variants found in a series of antibacterial *TLR* genes in Czech Simmental cattle and related populations demonstrates sufficient diversity in the production population and rejects the possibility of negative impacts from preferential breeding for production traits. The potential contribution of the conserved genetic resources to future resistance breeding is rather moderate in this particular case and it is restricted to the non-overlapping parts of diversity. An open question is whether this observation is transferable to other rare historical breeds of cattle. The located non-synonymous single-nucleotide polymorphisms with predicted and/or previously reported effects on the innate immunity system are expected to be used as genotyping targets in association studies and breeding projects aimed at the Simmental-type breeds.

## Data Availability

The complete list of identified SNPs has been deposited in the Open Science Framework data repository (, Novák, 2019). Additional data are available upon request to the corresponding author.
